# Marine Bioactive Compounds Derived from Macroalgae as New Potential Players in Drug Delivery Systems: A Review

**DOI:** 10.3390/pharmaceutics14091781

**Published:** 2022-08-25

**Authors:** Bogdan-Stefan Negreanu-Pirjol, Ticuta Negreanu-Pirjol, Dan Razvan Popoviciu, Ruxandra-Elena Anton, Ana-Maria Prelipcean

**Affiliations:** 1 Faculty of Pharmacy, Ovidius University of Constanta, 6, Capitan Aviator Al. Serbanescu Street, Campus, Corp C, 900470 Constanta, Romania; 2 Biological Sciences Section, Romanian Academy of Scientists, 3, Ilfov Street, 050044 Bucharest, Romania; 3 Faculty of Natural Sciences and Agricultural Sciences, Ovidius University of Constanta, 1, University Alley, Campus, Corp B, 900527 Constanta, Romania; 4 Cellular and Molecular Biology Department, National Institute of R&D for Biological Sciences, 296, Splaiul Independentei Bvd., 060031 Bucharest, Romania

**Keywords:** bioactive compounds, marine macroalgae, drug delivery, ulvans, fucoidans, carrageenans

## Abstract

The marine algal ecosystem is characterized by a rich ecological biodiversity and can be considered as an unexploited resource for the discovery and isolation of novel bioactive compounds. In recent years, marine macroalgae have begun to be explored for their valuable composition in bioactive compounds and opportunity to obtain different nutraceuticals. In comparison with their terrestrial counterparts, Black Sea macroalgae are potentially good sources of bioactive compounds with specific and unique biological activities, insufficiently used. Macroalgae present in different marine environments contain several biologically active metabolites, including polysaccharides, oligosaccharides, polyunsaturated fatty acids, sterols, proteins polyphenols, carotenoids, vitamins, and minerals. As a result, they have received huge interest given their promising potentialities in supporting antitumoral, antimicrobial, anti-inflammatory, immunomodulatory, antiangiogenic, antidiabetic, and neuroprotective properties. An additional advantage of ulvans, fucoidans and carrageenans is the biocompatibility and limited or no toxicity. This therapeutic potential is a great natural treasure to be exploited for the development of novel drug delivery systems in both preventive and therapeutic approaches. This overview aims to provide an insight into current knowledge focused on specific bioactive compounds, which represent each class of macroalgae e.g., ulvans, fucoidans and carrageenans, respectively, as valuable potential players in the development of innovative drug delivery systems.

## 1. Introduction

### The Diversity of Black Sea Macroalgae Species and Correlation with Their Medical Potential

Ocean or marine life includes numerous and varied organisms inhabiting marine water or coastal regions and, due to its remarkable biodiversity, it has become a valuable source for different natural resources, including many biologically active compounds [1]. The Black Sea coastal zone is of special interest due to a specific characteristic, being an area with a high amount of bioactive compounds, as the parameters of the abiotic environment show variations that are both dynamic and unpredictable (wind intensity and direction, storms, temperature variations, periods of isolation etc.). It is an area located at the interface between the sea and land, including surface and underground coastal waters, and also the adjacent lands influenced by the nearby shoreline), islands and salt lakes, wetlands adjacent to the sea, beaches, and cliffs [2].

Macroalgae are a diverse and polyphyletic group, encompassing different types of organisms capable of performing photosynthesis and featuring more or less differentiated tissues. The three main constitutive taxonomic groups are Chlorophyta (known as “green algae”), Rhodophyta (red algae), and Ochrophyta (especially Phaeophyceae class—brown algae). While there are around 297 species residing in the Black Sea, some of them are present in small populations or inhabit only limited areas of the sea, especially along the Bulgarian and Turkish coasts [3].

Some of the most common species are mentioned in Table 1.

**Figure 1:**
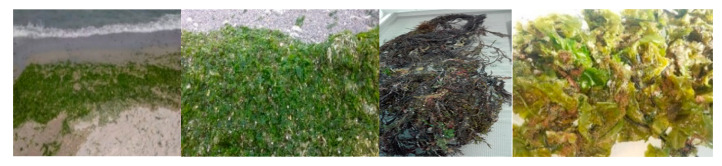
Green, brown, and red macroalgal biomass deposits along the Romanian Black Sea Coast (*Ulva*, *Cladophora*, *Cystoseira*, and *Ceramium* species) [2,6].

The frequency and length in time of episodes causing algal blooms deposited in high amounts is higher in the southern sector of Romanian Black Sea Coast is generated by the presence of underwater limestone platforms (Figure 1).

This macroalgal bloom is mostly encountered in the pre-vernal or vernal seasons or after storms [2]. Seaweeds (macroalgae) are well-known sources of bioactive compounds due to their complex habitat and exposure to extreme conditions. Thus, these bioactive compounds, while acting as valuable nutrients, also exhibit potent biological activities. For example, polysaccharides and peptides isolated from various marine sources exhibit antioxidant, anticancer, anticoagulant, and antidiabetic activities. 

Numerous studies addressed the biotechnological and medical potential of marine algal deposits along the Romanian seaside [5].

Marine macroalgae are an important resource, rich in dietary fiber, proteins, vitamins, and minerals. They also feature a wide array of secondary metabolites with valuable biological activities, such as proteins, lipids, carbohydrates, and carotenoids, some of which are unique when compared to terrestrial plants [4]. Hence, the potential application of macroalgae as a source of bioactive compounds with health benefits is of growing interest. Algal polyphenols, especially phlorotannins (polyphloroglucinols) found in brown algae, have been emphasized to have exceptional antioxidant properties. Furthermore, algal sulfated polysaccharides, carotenoids such as astaxanthin and fucoxanthin, sterols, peptides found in the structure of algal proteins, and mycosporine-like amino acids (MAAs) emphasize different ratios of antioxidant potential [7].

It is well-known that marine life forms constitute a global biodiversity, reflected also in the abundance of valuable natural bioactive compounds including, various amino acids, peptides, proteins, fatty acids, sterols, vitamins, oligosaccharides, and minerals. Thousands of new compounds are being isolated from marine organisms each year, further supporting the development of new drugs to diagnose or treat human diseases including cancer, inflammatory illnesses, and viral diseases [8].

Macroalgal tissue extracts can have various pharmacological uses. Until now, around 83 species worldwide were proven to be pharmacologically effective (49 red algae, 20 brown algae, and 14 green algae). Their applications are extremely varied; some act as antiviral (against colds, influenza etc.), some as antibacterial (e.g., against tuberculosis), and some act as antifungal agents.

*Cladophora* species contain a wide array of phytochemicals, including antibiotic and antitumoral agents, such as bromoascochlorins, polyunsaturated fatty agents and α-tocopherol (a strong antioxidant), valuable micronutrients (iron, magnesium, manganese, potassium, and zinc), and proteins. Aqueous extracts of *Cladophora vagabunda* macroalgae can improve blood health under stressful conditions [4].

*Ulva* species contain compounds that stimulate cardiovascular and nervous activity, act as antibiotic, diuretic, hypoglycemic, and contraceptive agents. Immunity-enhancing bromoascochlorins and polyunsaturated fatty acids are also present, together with vitamin B (mostly B_12_) and vitamin F, essential aminoacids, folic acid, and nicotinic acid. *Ulva (Enteromorpha) intestinalis* aqueous extracts are known as hepatoprotective. *Ulva linza* and *Ulva prolifera* are also a source of antioxidants and cytostatic polysaccharides.

Among brown algae, *Cystoseira barbata* acts as a source of alginates, amino acids, microelements (bromine, calcium, chlorine, iodine, magnesium, potassium, selenium, and sulfur), antitumoral (bromoascochlorins and polyunsaturated fatty acids), and antibiotic compounds.

## 2. Representative Delivery Systems Developed Based on Bioactive Compounds from Brown, Red, and Green Macroalgae Species

### 2.1. Phaeophyceae—Brown Algae 

#### 2.1.1. Fucoidan

Fucoidans are sulphated polysaccharides, contained in seaweeds and were first identified in brown algae in 1913 [9]. The fucoidans’ biochemical structural formula is based on sulphate groups or uronic acids bonded to L-fucose repetitive units linked by α-1-2 and α-1-4 bonds [10]. Based on the type of sugar, there are: U-fucoidan—contains glucuronic acid, F-fucoidan—includes only sulphated fucose and G-fucoidan—contains galactose [11]. Various brown algae such as *Saccharina japonica*, *Fucus vesiculosus*, *Undaria pinnatifida*, and *Hizikia fusiformis* but also from marine invertebrates such as sea cucumber were reported as fucoidan’s sources. The fucoidan’s content depends on the species, the geographical area, and the season in which it is harvested. Additionally, the environmental factors, such as salinity and nutrients, play important roles in determining the total content of the bioactive molecules [12].

Fucoidan is the king of biologically active compounds, highly branched, and dissimilar in monosaccharide composition with a large molecular weight (10,000–100,000 Da). The therapeutic potential is supported by the diverse chemical structure and the strong antioxidant activity. The mechanisms of action are operating via a wide range of pharmacological effects such as anti-inflammatory [13], antiangiogenic [14], anticoagulant [15], immunomodulatory [16], anti-adhesive [17], antitumoral [18], antidiabetic [19], antimicrobial [20], and anti-neurodegenerative [21]. 

Drug delivery systems are formulations or devices that grant access of a therapeutic agent into the human body (Table 2). Nanotechnology provides novel tools for improved drug delivery in terms of efficacy and safety by a more precise control of the rate, time, and location of the drug release. The main properties of a nanodelivery system are: *(i)* controlled drug release, *(ii)* ability to use different routes of administration, *(iii)* improved safety and efficacy of drugs, *(iv)* increased solubility, and *(v)* new market opportunities to recover drugs that failed at conventional delivery. Nevertheless, the main challenges refer to deficiencies in reaching the target site, in vivo instability, limited bioavailability, solubility, and absorption. Nanoparticle carriers are, for example, efficient drug delivery systems that have the ability to overcome these limitations [22].

#### 2.1.2. Drug Delivery Systems with Antiproliferative Potential

Fucoidan is a perfect choice when developing new drug delivery systems due to the numerous and various biological properties, thus extending the drugs targets. A study by Cavalcanti et al. explored different activities of incorporated oncocalyxone A within nanoparticles of isobutyl cyanoacrylate coated with fucoidan. Haemolysis, means the damage the red blood cells and in this way the haemolytic processes were not registered for any of the tested nanoparticle concentrations, up to the highest one, 330 µM, respectively. In addition, the antiproliferative effect was observed in vitro for the following concentrations, 25 µg/mL and 50 µg/mL on MDA-MB-231 cancer cell line. Furthermore, no cytotoxic effects were registered on normal macrophages J774A.1. Their findings showed a promising way to deliver oncocalyxone A while ensuring a reduced toxicity via the fucoidan coating [23].

Jafari et al. reported the development of a fucoidan-based drug delivery system for an efficient breast cancer therapy via fucoidan-doxorubicin nanoparticles. In 2020, there were 2.3 million women diagnosed with breast cancer and 685,000 deaths globally. Metastatic breast cancer is the most prevalent cancer affecting both women and men, with a survival chance of 5–10 years. Metastasis is possible via the interaction with blood constituents, such as platelets, leukocytes, and selectins that were identified as key adhesion molecules. P-selectins are crucial actors in metastasis because of the cancer cells attachment to the endothelium and platelet activation in distant organs. The in vitro results on two breast cancer cell lines, MDA-MIB-468 and MDS-MIB-231, showed a higher efficiency on MDA-MB-231 cells due to the specificity towards P-selectin expression. The nanoparticles obtained by direct conjugation of doxorubicin to the fucoidan backbone proved to be a robust platform for controlled doxorubicin release. The promising approach aims to maximize the targeting competence and to limit the toxicity of doxorubicin [24]. Chiang et al. highlighted the potential of fucoidan nanoparticles in cancer therapies. Their research demonstrated the greater antiproliferative activity of fucoidan nanoparticles in vitro for 0.5 mg/mL on the MDA-MB-231 cancer cell line when compared to fucoidan only. The results were validated in an in vivo model; in addition, the study investigated fucoidan intravascular delivery. The intraperitoneal injections that have been administrated to groups of mice at different concentrations reported atypical actions for one of these groups. Among the tested concentrations (0 to 500 mg/kg of fucoidan), only the high dose group of 500 mg/kg showed anomalies; the histological examination revealed modifications solely at the lymphatic organ level [25].

#### 2.1.3. Drug Delivery Systems with Antimicrobial Potential

Antibiotic resistance in bacteria arises by gaining changes in their genes as a defensive mechanism. With the increasing number of resistant micro-organisms against drugs that once proved to be protective, there is a high need for novel anti-infectious agents. 

Oka et al. showed that fucoidans obtained from *Fucus vesiculosus* and *Cladosiphon novae-caledoniae* exhibit strong antimicrobial activity, which was comparable to fluconazole against *Candida albicans*. Moreover, the marine fucoidans were efficient against oral pathogens e.g., *Streptococcus mutans* and *Porphyromonas gingivalis* by interfering with the bacterial growth and impairing the adhesion to bovine teeth [26]. Alghuthaymi et al. tested the activity of metal (Ag and Se) and fucoidan-based nanoparticles against three fungal strains, *Candida albicans*, *Candida glabrata*, and *Candida parapsilosis*, respectively [27]. Fucoidan, obtained from brown algae *Cystoseira barbata*, was used as a stabilizing agent and also as a bioactivity and biocompatibility potentiator. The antifungal activities of both types of nanoparticles were stimulated by the use of fucoidan-based biopolymer matrix that ensured nanoparticle stabilization, distribution, and ability to adhere to yeast cells via membrane interaction. 

In addition, Rao et al. reported strong antibacterial properties of Ag nanoparticles using fucoidan from *F. vesiculosus* against the pathogenic strain *Pseudomonas aeruginosa*; this was mainly due to the fucoidan-nanoparticles’ interaction with microbial cells and the consequential leakage of intracellular proteins and ROS oxidative stress [28]. Furthermore, a biofilm inhibitory concentration was established at 20 mg/mL. 

Furthermore, Shanti et al. reported the synthesis of anionic Ag nanoparticles encapsulated in fucoidan obtained from *Turbinaria decurrens* with strong antibacterial activity against Gram-negative clinical pathogens. The functional groups -OH, -CH, and S=O associated with sugar residues in the sulfated fucoidan are involved in the synthesis of spherical nanoparticles in the range of 10–60 nm with polydispersity and a tendency to aggregate [29].

#### 2.1.4. Drug Delivery Systems with Anti-Inflammatory Potential

In general, the inflammation occurs in response to damage and in that way, the immune system is stimulated to become a stronghold against harmful agents. The inflammatory response can lead to a destructive mechanism of the tissues, and the inflammation to chronic diseases or necrotic cells. Fucoidan is known for its anti-inflammatory actions and in their study, El-Far et al., investigated its effect in an animal model. The rodents have been divided into five groups and three of them have been force-fed with 100 mg/kg or 200 mg/kg of fucoidan in a hepatocellular carcinoma model. Their results showed encouraging data as fucoidan intake led to better survival rates and displayed hepatoprotective activity suggested by the low serum level of alfa-fetoprotein. The outcome was a lowered expression of miR-143, NF-κB, and of proinflammatory cytokines TNF-α and IL-1β in rats [30].

In order to increase quercetin bioavailability and stability, Barbosa et al. developed polymeric nanoparticles based on fucoidan and chitosan as an oral drug delivery system. The quercetin-loaded fucoidan/chitosan nanoparticles obtained through the polyelectrolyte self-assembly method proved to exhibit strong antioxidant properties [31]. In addition, a controlled release in simulated gastrointestinal environments was observed, in particular, for the variants with higher fucoidan content (3:1 and 5:1 fucoidan/chitosan ratios), with prevention of quercetin degradation and an increase of its oral bioavailability. Similar polymeric nanoparticles fucoidan/chitosan were analyzed as promising and non-invasive transdermal drug delivery systems of methotrexate for improved treatment management of skin diseases [32]. Drug0direct application elicits delivery to the dermal microcirculation, thus stimulating molecular inflammatory storms. Therefore, specific permeability is a highly valued property in the development of the carrier approaches. Nanoparticles in the range of 300–500 nm registered an entrapment efficiency >80%, positive zeta potential for 1:1 fucoidan/chitosan, and negative surface charge for 3:1 and 5:1 ratios. The methotrexate-loaded fucoidan/chitosan nanoparticles when compared to free methotrexate improved *(i)* cytocompatibility in in vitro fibroblast and human keratinocytes cultures and *(ii)* skin permeation in an in vitro pig ear skin model. Moreover, the in vitro pro-inflammatory cytokine production was significantly reduced. 

Fucoidan has also shown therapeutic potential in the case of liver diseases, which are linked to various factors such as pathogens, drugs or alcohol excess, and genetic predisposition. Zhao et al. propose fucoidan as a safeguard to alleviate ethanol-induced hepatic lesions in male rats [33]. Fucoidan pre-treatment (150 and 300 mg/kg body weight) provided protection against ethanol-induced oxidative stress and further restored the mitochondrial function leading to reduced mitophagy and apoptosis. Thus, the results recommend the potential of fucoidan for alcoholic liver diseases prevention and treatment.

#### 2.1.5. Drug Delivery Systems with Antidiabetic Potential

Type II diabetes mellitus is a global chronic metabolic disorder, reported to pose a serious threat to human health. Peng et al. advanced an innovative approach in order to ameliorate hyperglycaemia and diabetic nephropathy in a type II diabetes mellitus Wistar rat model, a combination of fucoidan and a traditional Chinese medicine formula [34]. The treatment with the therapeutic combination resulted in decreased blood glucose, insulin resistance, serum lipid, and antioxidant stress levels. Furthermore, the synergistic combination stimulated the hypoglycaemic activity by interfering with the glucose metabolism pathway. Additionally, it significantly improved renal function and structure, providing an efficient alternative to diabetes mellitus treatment. Furthermore, Daub et al. target in their study the implications of enzyme inhibition, α-amylase and α-glucosidase, respectively. Both enzymes are involved in dietary starch metabolism, leading to increased blood glucose levels. The study’s purpose was to test the potential inhibitory effects of fucoidan on the enzymatic activity with consequent control of blood glucose. Fucoidan obtained from *Ecklonia maxima* significantly inhibited α-glucosidase enzyme activity in the range of 0.1–1 mg/mL and had similar results to the commercial fucoidan from *Fucus vesiculosus* [35].

**Table 2:** Summary of fucoidan and the delivery systems used for various bioactive properties valorization.

Bioactive Property	Drug Delivery System	Mechanism of Action	Reference
Antitumoral	Oral administration of fucoidan extract	Suppression of tumor in vivo mice model	[36]
	Oral administration of 100 mg/kg fucoidan extract	Inhibition of tumor growth in vivo mice model	[37]
	Agar matrix mix of fucoidan extracts from three algae	Inhibition of SK-MEL-28 human melanoma cells and DLD-1 colon cancer cells	[38]
	Purified fucoidan extract	Inhibition of colony formation of DLD-1 cancer cells	[39]
	Fucoidan extract	Induce cell apoptosis in B16 murine melanoma cells	[40]
	Fucoidan extract	Inhibits DU-145 human prostate cancer cells migration and hiders tumor growth in cancer xenograft	[14]
	Fucoidan extract	Induce apoptotic cell death in HCT116 human colorectal carcinoma cells	[41]
	Intraperitoneal injection of fucoidan	Inhibits tumor growth and induce apoptosis in 4T1 tumor bearing Balb/c mice	[42]
	Oral administration	Hinders metastasis in Lewis tumor-bearing mice	[43]
	Fucoidan extract as potential anticancer agent	Inhibits HT-29 human colon adenocarcinoma cells	[44]
	Oral administration	Inhibits tumor growth in LLC1-bering mice	[45]
Antioxidant	Purified fucoidan extract	High antioxidant activities due to high sulfate content in new extraction procedure	[46]
	Fucoidan extract	Presents strong scavenging activity and could be used as natural antioxidant in diseases treatments	[47]
Immune-modulatory effect	Intraperitoneal injection	Up-regulates CD40, CD80, CD86, MHC class I and MHC class II in spleen dendritic cells	[16]
	Intraperitoneal injection of fucoidan	Enchanced Natural Killer cells activity in spleen of C57BL/6 mice	[48]
Anti-inflammatory	High-molecular-weight product of fucoidan (150 mL/day)	Reducing toxicity in patience going thru chemotherapy	[49]
	Fucoidan extract—Mei Han Yun product	Stimulates natural immunity	[50]
	Dietary supplement	Inhibits atopic dermatitis skin lesions and immune system abnormalities	[51]
	Fucoidan extract	Reducing toxicity and inhibition of reactive oxygen species and nitric oxide generation	[52]
	Fucoidan extract	Inhibition expression of IL-1B, IL-6, TARC and MDC in TNF-α/IFN-γ induced HaCaT human keratinocyte cell line	[53]
	Oral and parenteral administration of fucoidan	Hypocholesterolemic effect and reduce inflammation	[54]
	Fucoidan administered as adjuvant	Induce pro-inflammatory cytokine production from spleen in C57BL/6 mice Enhances antigen presentation and antigen specific T cell proliferation in C57BL/6 mice	[55]
	Treatment with purified fucoidan extract	Inhibition of nitric oxide production in LPS-exposed zebrafish embryos	[56]
	Oral administration of low molecular weight fucoidan	Down regulate expression of IL-6 and up-regulate IL-10 in apoE-knockout mice	[57]
	Topical application of lyophilized fucoidan powder	Improve symptoms of atopic dermatitis in AD-induced Nc/Nga mice	[58]

### 2.2. Rodophyta—Red Algae

#### Carrageenans

Carrageenans are linear sulphated polysaccharides and are encountered in various red algae genera from the *Florideophyceae* class such as, *Furcellaria*, *Agardhiella*, *Chondrus*, *Eucheuma*, *Hypnea*, *Iridaea*, *Solieria*, *Sarconema*, and *Gigartina* [59,60]. Carrageenans are generally recognized as safe (GRAS) by the Food and Drug Administration (FDA) since 1973. Carrageenan (E-407) and semi-refined carrageenan (E407a) have been approved by the European Food Safety Authority as food additives. In terms of toxicity, carrageenans express minimal or no adverse physiological effects [61]. In recent years, a growing interest has been oriented towards carrageenans due to a series of advantageous characteristics such as biocompatibility, high molecular weight, high viscosity, and gelation. 

**Figure 2:**
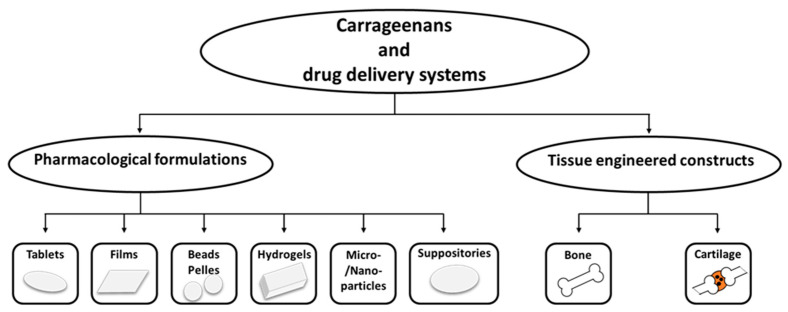
Carrageenans and representative drug delivery systems, (adapted from [63]).

The potential applications have widened to the medical, pharmaceutical, and bio-technological fields; an important number of research papers are focusing on this type of polysaccharide (Table 3). Today, carrageenans represent one of the main biomaterials for a wide span of applications in the pharmaceutical industry due to their ability to improve drug formulation and their controlled delivery and release [62,63], Figure 2.

Carrageenans are a group of high-molecular-weight hydrophilic sulphated polysaccharides constituted from alternative units of D-galactose and 3,6-anhydro-galactose (3,6-AG) joined by alternating α-1,3 and β-1,4-glycosidic bonds. It is an anionic polysaccharide due to the 15–40% content of ester-sulphate. According to their sulphate content, the source of isolation and solubility, carrageenans can be grouped into six basic forms: kappa (κ-), iota (ι-), lambda (λ-), mu (μ-), nu (ν-), beta (β-), and Theta (θ-) carrageenan. The representative types with applications in the pharmaceutical industry are kappa-, iota-, and lambda- carrageenan. Given that the majority of seaweeds contain mixed carrageenans, usually, for the isolation a well-defined mixture is obtained [60]. The difference between these three types is given by the number of ester-sulphate groups per repeating disaccharide unit, respectively, one (kappa-), two (iota-), and three (lambda-). The biochemical structures of carrageenans are characterized by heterogeneity and correlated to the algae sources, the extraction procedure, and the algal life stage [64].

When making hydrogels, the most important characteristic is pore size, ensuring that the specific drug to be delivered will be ready to be entrapped within its structure and then released when needed. In a study by Sharifzadeh et al., they followed the most important properties in hydrogels, made with *κ*-carrageenans, and studied the swelling, drug loading/release, and cytotoxicity. The results showed the non-toxicity of hydrogels and a high release percentage, respectively, 68.28% and 59.21% of rhodamine B (RB) model drug, when pure κ-carrageenan hydrogel was used in vitro drug release studies [65].

Pettinelli et al. advanced a composite hydrogel as a novel dual drug delivery system. In the hydrogel based on kappa-carrageenan and locust bean gum, poly hydroxybutyrate-co-hydroxyvalerate microparticles containing ketoprofen and mupirocin were enclosed [66]. Diffusion was the main mechanism for release of both drugs encapsulated in spherical microparticles with a mean size of 1µm and homogenously distributed in the hydrogel. A strong hydrogel was obtained which was confirmed by the ratio of elastic/viscous components. In addition, the biocompatibility of the construct was validated in vitro on an embryonic fibroblast cell culture, thus recommending the composite hydrogel as a potent delivery carrier of poorly water-soluble drugs with applications in the wound healing field. 

For similar medical applications, Fahmy et al. prepared a novel bioactive film based on kappa-carrageenan, sodium alginate, and cross-linked with potassium ions [67]. The swelling and the gel fraction properties confirmed the potential for wound healing applications. The obtained film expressed tunable properties, silver nanoparticles being added in order to enhance the antibacterial potential against both Gram-positive and Gram-negative strains. 

Furthermore, study of Joshi et al. aimed to obtain a polyelectrolyte complex hydrogel using kappa-carrageenan-montmorillonite with extended-release potential [68]. The interactions formed during the gelation process between the cationic silicate hydroxylated edge groups of montmorillonite and the anionic sulphate group of kappa-carrageenan, were confirmed by FTIR analysis. The pellets obtained by the extrusion–spheronization procedure lead to the in vitro drug release (tapentadol) up to 12 h, recommending the formulation as an alternative to conventional pain management agents. 

Youssouf et al. used kappa-carrageenan, extracted from *Kappaphycus alvarezii* (elkhorn sea moss) and polycaprolactone, in order to develop nanomicelles for hydrophobic drug delivery; the model-drug used was curcumin [69]. The oligocarrageenans-based nanomicelles were biocompatible as evaluated in vitro on EA-hy926 endothelial cells and confirmed in vivo on zebrafish. Additionally, strong anti-inflammatory activity was observed, 15 µM encapsulated curcumin revealed an absolute inhibition of TNF-alpha-induced inflammation when comparing it to the free form. Thus, these promising results support the potential use of the polycaprolactone-grafted oligocarrageenan composite as a suitable delivery system for hydrophobic compounds to different organs.

Vinothini et al. employed kappa-carrageenan in the development of a carrier system targeting cervical cancer treatment, a severe type in the female population [70]. The complex was obtained via the synthesis of kappa-carrageenan grafted graphene oxide nanocarrier conjugated with biotin. The drug tested for entrapment was doxorubicin, a known antitumoral agent. Human cervical cancer cells, HeLa, suffered reduced cell viability and proliferation, apoptosis induction, and nuclear chromatin condensation, suggesting a robust potential of the antitumoral platform. 

The biochemical particularity of carrageenan was exploited by Rodrigues et al. in order to design carrageenan-based microparticles for an inhalable tuberculosis treatment [71]. Infectious pathogens present a series of moieties that are preferentially recognized by macrophage receptors; carrageenans are benefitting from a similar composition, which suggests they may be perfect candidates for drug carriers and targeting macrophages receptors. Inhalable starch/carrageenan microparticles were associated with isoniazid and rifabutin and showed no cytotoxic effects on lung epithelial cells (A549) while activating macrophage target cells (via IL-8 secretion). The study proved to be an innovative approach for inhalable drug carriers. 

Biofilm infections are serious threats to various fields from food packaging to biomedical applications. Microbial biofilms are physiologically distinct from their planktonic version, with a well-defined structure that possess increased resistance to environmental stress or drugs and are hard to eradicate. There is an urgent need for innovative and emergent solutions to fight problems associated with biofilm development and maturation. For instance, a synergistic association of kappa-carrageenan and silver nanoparticles showed tremendous potential in inhibiting both *Staphylococcus aureus* and *Pseudomonas aeruginosa* mediated biofilms [72]. The nanocomposites maintained the excellent thermal stability and antibacterial potential when encapsulated in crosslinked hydrogel, providing promising platforms for antibacterial biofilms.

**Table 3:** Summary of carrageenans and the delivery systems used for various bioactive properties valorization.

Bioactive Property	Drug Delivery System	Use	Reference
Antiviral activity	Extracts for drug formulation, acting as inhibitors for viruses	Holds back Human Rhinoviruses, Herpes Simplex Virus (HSV), Varicella Zoster Virus (VZV) and Human Papillomavirus (HPV)	[73,74,75,76,77,78]
	Novel core-matrix intravaginal ring	Inhibits HPV and HSV-2	[79]
	Gel formation—*Carvir*	Bioactive activity against HPV infection	[80]
	Iota-carrageenan nasal-spray	Ameliorate cold symptoms and inhibits the multiplication of Human Rhinoviruses (HRV)	[81]
	Intranasal iota-carrageenan	Could hold back the Influenza A Virus infection	[82]
	κ-carrageenan extract specific targeting	Inhibits H1N1/2009 and other similar viruses	[83]
	Intranasal application synergy of carrageenan and zanamivir	Holds back Influenza A Virus strains (pandemic H1N1/09, H3N2, H5N1, H7N7	[84]
	Nasal spray	Inhibits Human Rhinovirus (HRV) 1a, hRV8 and Human Coronavirus OC43	[85]
	κ-carrageenan extract in plaque reduction assay	Bioactive activity against Enterovirus 71 (EV 71)	[86]
	λ-carrageenan P32 extract as promising drug	Bioactive activity against Rabies Virus (RABV)	[87]
	Polysaccharide carrageenan extract	Bioactive activity against Varicella Zostre Virus (VZV)	[88]
	Polysaccharide carrageenan extract	Bioactive activity against Severe acute respiratory syndrome coronavirus 2 (SARS-CoV-2)	[89]
Antibacterial activity	Polysaccharide carrageenan extract suggested to act as preservatives in processed food	Acts against the growth of different bacterial strains	[90]
	Local application of iota-carrageenan	Bioactive activity against the ocular infection caused by *Chlamydia trachomatis*	[91]
	k-carrageenan oligosaccharide extract	Is hostile for *Saccharomices cerevisiae*	[92]
	Oxidized κ-carrageenan	Inhibits the growth of Gram-positive and Gram-negative bacteria	[93]
	Carrageenan added in sinus rinses	In the presence of Kappa-Carrageenan cells release less IL-6	[94]
	Hydrogel formation	Inhibits the growth of *Staphyloccocus aureus* and *Escherichia coli*	[95]
	Carboxymethylation of κ-carrageenan for biomaterial applications	Inhibits the growth of *Bacillus cereus*, *Pseudomonas aeruginosa*, *Staphyloccocus aureus* and *Escherichia coli*	[96]
Antihyperlipidemic activity	Viscous gels	Lowering blood levels of cholesterol	[76]
	Seaweed powder mix as health supplement Carrageenan microgels	Lowering serum levels of triglycerides, low density lipoprotein cholesterol (LDL-C) and total cholesterol, and raises levels of high-density lipoprotein cholesterol (HDL-C)	[97,98]
	Carrageenan extract as supplement	Regulate prostaglandin E2 synthesis and stimulate IL-1 and IL-6 synthesis. Cholesterol reducing properties	[99]
	Gel formation for vegetable ingestible jelly	Lowering serum levels of total cholesterol	[100]
	Carrageenans as food supplement and prebiotics	Bioactivity in metabolic syndrome	[101,102]
Anticoagulant and antithrombotic activity	Extracts	Most effective anticoagulant tested on rabbits	[103]
	Carrageenan as excipient	Reduces formation of blood clots	[62,73]
	Synthesis of carrageenan derivatives	Acts as an anticoagulant	[104]
Antitumor and immunomodulatory activity	Adjuvants Synthesis of carrageenan oligosaccharide derivatives	Acts as an immunomodulator with anticancer effects	[105,106]
	Extract of low molecular weight λ-carrageenan	Increases the antitumor effect of 5-Fluorouracil	[107]
	λ-carrageenan intratumoral injection	Holds back the growth of tumors in mice with murine melanoma cell line	[108]
	Extract as anticancer agent	*Kappa*-CG and *Lambda*-CG delays the cell cycle in the G2/M phase, while only the last stalles the cell cycle in the G1 and phase	[109]
	Degraded iota-carrageenan	Holds back tumor growth, can induce apoptosis, and stop the G1 phase	[110]
	Active principles of extracts	Damages LM2 tumor cells	[111]
	Extracts	Inhibits colorectal cancer stem-like cells	[112]
	LMW carrageenan degradation products	Modulates the immune system with anticancer effects	[113]
Antioxidant activity	Multilayer coating based on κ-carrageenan and quercetin-loaded lecithin/chitosan nanoparticles	Antioxidant activity in the multilayer coating	[114]

**Figure 3:**
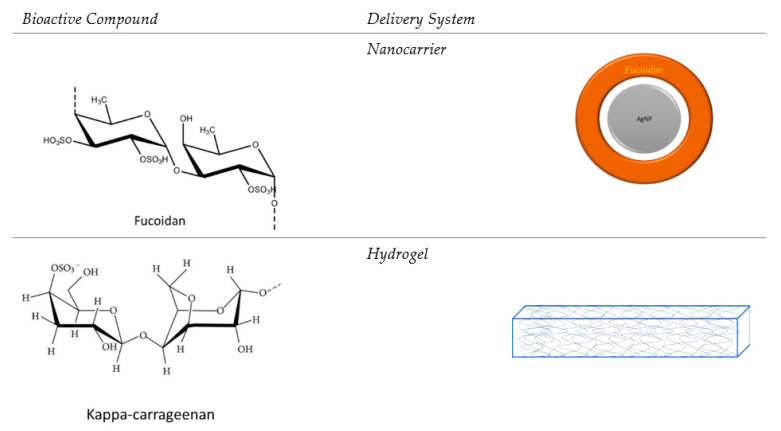
The bioactive compounds and the most representative delivery systems.

In the Figure 3, is presented the important bioactive compound from brown algae and red algae, refers to fucoidan and kappa-carrageenan and the representative delivery systems.

### 2.3. Chlorophyta (Green Algae)

#### 2.3.1. Ulvans

Green macroalgae Ulvales (Chlorophyta), namely *Ulva* sp. and *Entermorpha* sp., genera are distributed worldwide and also on the Romanian shoreline of the Black Sea [115,116]. The macroalgae cell wall matrix contains molecules of the gelling sulphated complex of heteropolysaccharides named ulvans, a water-soluble sulphated polysaccharide, which represents about 8–29% of the dry weight algae mass [117]. Ulvan as sulfated polysaccharides is the main component of the cell wall of the green algae belonging to the *Ulva genus* [118,119]. This complex, which represents 38–54% of the dry mass, consists of different sugar residues depending on the macroalgae strain and is mainly composed of sulfated L-rhamnose, D-glucuronic acid and its C5-epimer L-iduronic acid (as found in animal glycosaminoglycans), and a minor fraction of D-xylose [120,121,122].

Regarding ulvan extraction, from the cell wall green macroalgae, one simple method is performed with hot water (80–90 °C) in the presence of a divalent cation chelator (ammonium oxalate) [123]. Then, purification is realized by alcohol or quaternary ammonium salt precipitation [121,124]. The extraction method, as reviewed by [125], could influence the structure of ulvan. Acidic conditions and the presence of a calcium-sequestering agent, were reported to reduce the physical interactions between the polymeric aggregates within the algal cell walls and favor the yield of ulvan extraction [126,127].

Ulvan, a sulfated polysaccharide, is mainly composed of l-rhamnose, rhamnose 3-sulfate, d-xylose, xylose 2-sulfate, d-glucose, and d-glucuronic acid, in a linear arrangement [120,128]. In addition, glucuronic acid and rhamnose are under the form of aldobiuronic acid (4-o-β-d-glucuronosyl-l-rhamnose) and iduronic acid [129]. Small amounts of sulfated groups are found in position two of the xylose residue, while most of them are located in position three of the rhamnose unit [121,130]. Some hydroxyl groups of the subunits are substituted by sulfate groups, while the borate group is associated with the rhamnose subunits, which do not contain any sulfate groups and calcium binds to borate, cross-linking the rhamnose groups. This borate–calcium complex leads to the rigidity of the cell wall [131].

**Figure 4:**
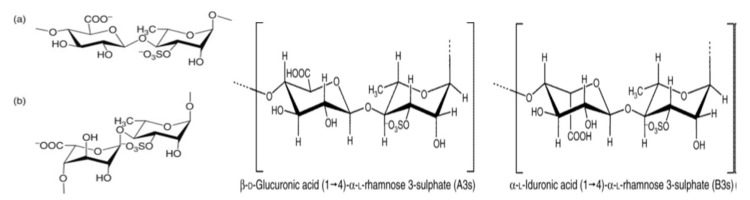
Structure of ulvanobiuronic acid units, (**a**) β-D-glucuronosyluronic acid-(1,4)-α-L-rhamnose 3-sulfate and (**b**) α-iduronopyranosic acid-(1,4)-α-L-rhamnose 3-sulfate [121,137].

As Figure 4 indicates, ulvan is divided into main ulvanobiuronic acid units represented by (a) (β-D-glucuronosyluronic acid-(1,4)-α-L-rhamnose 3-sulfate) and (b) (α-L-iduronopyranosic acid—(1,4)-α-L-rhamnose 3-sulfate) [121,129,132]. The molecular weight of this sulfated polysaccharide ranges from 150 kDa to 2000 kDa [133,134,135]. Hence, its chemical composition is complex and variable [136]. Due to its content of carboxyl, hydroxyl, sulfate—hydrophilic groups and methyl—hydrophobic groups, in the form of sulfated polysaccharide, ulvan has unique physicochemical properties.

Other components of green macroalgae cell walls are xylan, mannan, and cellulose. *Ulva lactuca* has been established as a better source of dietary fiber compared to fruits and vegetables and the insoluble part of dietary fiber is in the highest amount, formed especially by hemicellulose as the most abundant fraction (20.6%), followed by cellulose (9.13%) and lignin (1.56%) as NDF, according to [138]. Ulvan is considered a dietary soluble fiber, yet it is resistant to both human enzymes of the gastrointestinal tract and degradation by human colonic bacteria. 

Ulvans and ulvan-derived polysaccharides from green macroalgae *Ulva pertusa* could exhibit various biological activities, such as antioxidant activity, decreased total serum cholesterol and LDL cholesterol, and the reduction of triglycerides as significant risk factors in CVDs [139]. In addition, ulvan has several physico-chemical and biological characteristics of potential interest for chemical and pharmaceutical applications [121,140]. 

Macroalgae-derived sulfated polysaccharides (SPs) such as fucans, fucoidans, carrageenans, and ulvans have demonstrated an array of biological effects. The biological effects as an antioxidant, antiproliferative, immunomodulator, anti-inflammatory, anticoagulant, antilipemic, antiviral, antibacterial, antiprotozoan, osteoprotective, and antivenomic could put SPs in the forefront of pharmaceutical research [141]. However, given the structural attributes interact with host factors in different ways, the biological efficacy of SP varies widely via various processes.

It is known that a major contributor to the formation of cancer cells in the human body is the oxidative stress due to the formation of free radicals. A range of molecular traits can control specific activities and, in turn, specific molecular traits could contribute to a range of biological activities. Thus, low molecular weights could increase degree of sulfation in addition to the uronic acid content, which appear to increase anti-oxidant activity in the case of ulvan [142,143,144,145].

Several scientific papers have suggested that low molecular weight SPs have shown stronger antioxidant activity than high molecular weight SPs. An important contributor to carcinogenesis is oxidative damage and these SPs from marine macroalgae are known to be important free-radical scavengers and antioxidants [146]. Ulvans with different molecular weight, derived from *Ulva pertusa* (Chlorophyceae) were analyzed and the results, in terms of antioxidant activity showed that low molecular weight ulvans have strong antioxidant activity due to the ability to be incorporated into cells and donate protons more efficiently compared to high molecular weight SPs [143]. This antioxidant potential of the ulvans can be directed towards the preparation of medicinal compounds that could control the progress of cancer, immunomodulatory, and antitumor activity [147,148]. Furthermore, the application of ulvans may be based also on their biological action, such as anticoagulation [149], polar interaction. For example, between the SP and proteins (such as heparin cofactor II) is consider to be a responsible factor for the anticoagulant activity and antihyperlipidemic activity. It was suggested that ulvan binds bile acids, thus expressing antihyperlipidemic activity [150,151,152,153], hypercholesterolemia, diabetes, and the reduction of the risk of colon cancer [154].

Ulvan has shown antioxidant activity due to its radical scavenging and metal chelating properties. Qi et al., 2010, reported that ulvan shows a higher antioxidant activity in scavenging free radicals than vitamin C, which is conventionally used as an antioxidant [155].

El-Baky et al., 2009 and Qi and Sun, 2015, reported that the antiproliferative and antihyperlipidemic effects of the sulphated polysaccharide are supported by ulvan antioxidant activity [156,157]. The antitumor activity of the ulvan is due to the main structural elements, sulphate, and uronic groups [156]. The antitumoral activity of ulvan has been demonstrated in vitro and in vivo against different types of cell lines [147,158,159]. These preliminary studies regarding the ulvan antiproliferative activity indicated that sulphated polysaccharides could be used as promising and safe substitutes for obtaining synthetic drugs, conventionally used in cancer therapy. In addition, it has been reported, to have a wide variety of bioactivities including antibacterial, immunostimulating, and antiviral activities [160,161]. Consequently, it has great potential to human health, agricultural, and biomaterial products [162,163].

Regarding the anti-inflammatory activity, the proposed and proven routes are selecting the blockade, complement cascades, and enzyme inhibition [164].

The mechanism against the pathogenic viruses is based on the blockage of the viral adsorption and internalization [164,165]. This specific polysaccharide composition of green macroalgae, for example, uronic acids, are important factors in the binding of certain viruses. In addition, glucuronic acid is a specific molecule of cell membrane used by respiratory viruses for cell invasion and consequently, the incubation of cells with iduronic acid could prevent this invasion by blocking the viral glucuronic acid receptors [166]. In this way, ulvan is unique macroalgae regarding its glucuronic and iduronic acid composition.

Ulvan immonumodulatory properties have been reported in parallel with the stimulatory effects on macrophages exerted by the mammalian homologous GAGs [167]. Moreover, ulvan derived from *Ulva rigida* induced an increase in nitric oxide production in macrophage culture, an effect that is usually associated with host defense mechanisms against pathogen agents [167]. Furthermore, some other studies indicated that the SPs extracted from green algae *Ulva intestinalis* may be involved in activating the immune system by stimulating macrophage cells to produce nitric oxide [168].

Additionally, ulvan has interesting rheological properties allowing it to be considered as a food-grade polysaccharide. An acidic extract of ulvan (T = 80 °C and pH = 2) showed thixotropic behavior with a dense network and interconnected chains, suggesting a strong gel formation as shown by [169]. In addition, Shao et al., 2014, showed that diluted solutions of ulvan present a rod-climbing effect and achieved an cold-set gelation with the addition of no cation [170]. These results could lead to the conclusion that ulvan could be used as a suitable source of gelling polysaccharides. However, high amounts of uronic acid (~30%) will strongly influence the rheological properties of ulvan, and negatively affect the viscosity of ulvan [134].

Nevertheless, in the absence of controlled and suitable production of the polysaccharides from green macroalgae, efforts are made for chemical structure optimization towards commercially scaled and more consistent polysaccharides, such as the alginates. In this regard, understanding the factors that influence the variability of macroalgae sulphated polysaccharides will contribute to the development of traceable and quality-controlled production systems that can deliver active products.

In conclusion, SPs in the cell walls of marine macroalgae, for example ulvans in green algae, carrageenans in red algae, and fucoidans in brown algae, can contribute to various applications in the development of pharmaceuticals, nutraceuticals, medicines, and tissue engineering including wound healing and cosmetics [171].

#### 2.3.2. Targeted Delivery System

It is known that the efficiency of a targeted delivery system, natural or engineered, can be enhanced by optimization of both the extent and rate of absorption.

Delivery systems may also have considerable benefits by protecting bioactive components and preventing the loss of the component, or adverse interactions with absorption inhibitors.

Achieving optimum (amount, rate, and site) rather than maximum absorption via controlled or targeted delivery is particularly pertinent in understanding the bioavailability concept. 

The term ‘bioavailability’ has arisen in the field of pharmacology, where it is a useful working concept of the rate and extent to which a drug or a bioactive component reaches its site of action. Bioavailability includes number of components, absorption, distribution, metabolism, and excretion, with subsequent biochemical and physiological effects.

The pharmacological concept of this term refers to the site-specific drug or bioactive compound delivery in order to treat a disorder. 

The bioactive compounds from green macroalgae whose structure and chemical properties vary depending on the different algal origin, have been investigated more in the last decade. It is known that this marine biomass contains bioactive compounds and functional polymers [172], and the total polysaccharide concentration of the algal biomass composition can reach up to 75% of the dry weight [173]. These polymers, which represent the soluble fraction of the algae polysaccharides, are classified as dietary fibers, but cannot be digested by the human body. 

**Figure 5:**
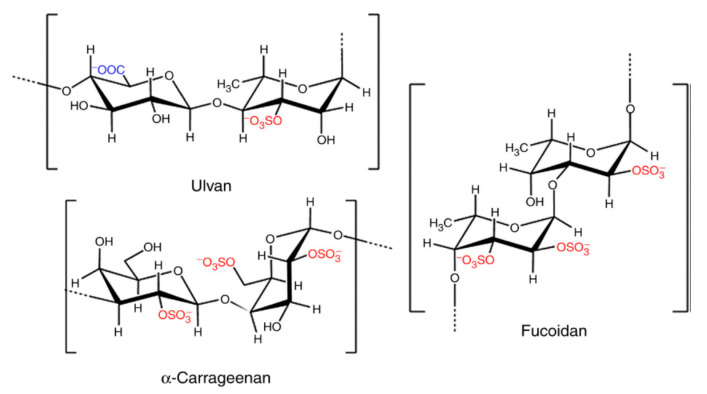
The structures of repeating units from the representative sulphated polysaccharides of macroalgal origin, adapted from [174,175]. In red color are highlighted the sulphated groups. In blue are the repetitive specific units (L-fucose, L-rhamnose and D-galactose).

The majority of the SPs’ biological properties have been assigned to the presence of sulphate groups (Figure 5) found in the algae cell wall matrix. In particular, the position of the sulphate groups present in the chemical structure of the polysaccharide has been shown to be decisive in providing the material with bioactivity [174].

Sulfated polysaccharides derived from macroalgae, are unique as biomaterials, due to their spectacular chemical versatility, excellent biocompatibility, and specific bioactivity, a unique combination, which is not easily found in any other chemical compound class [165]. 

In recent years, the main prospects for sulfated polysaccharides of algal origin have been the therapeutic replacement of mammalian GAGs to mimic their function as safer and cheaper alternatives to those of animal origins [176,177,178] and the preparation of polymeric matrices as drugs and/or cellular vehicles that naturally integrate into the native environment of GAGs [179].

**Figure 6:**
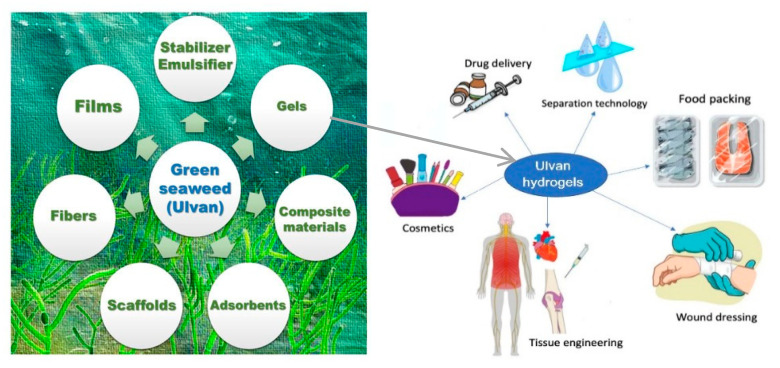
Possible bio-based materials using green seaweed (ulvan) polysaccharides, with some modifications, as a suitable source (adapted from [181,182]).

Despite the fact that it is the most abundant algal biomass and is involved in environmental processes named *green tides* [179,180], SPs obtained from green macroalgae belonging to *Ulva* species or *sea lattuce*, are much less investigated but could be considered the most promising macroalgae, Figure 6.

The development of biotechnological biorefining processes could transform this macroalgae from an under-exploited and polluting biomass into a valuable biomaterial resource [181,183].

The sulphated polysaccharide can be considered polymeric materials, acting as structural components of algae cell walls where they are bound covalently or weakly aggregated by electrostatic interactions, mediated by calcium ions [184]. 

In this respect, the chemical structure of ulvan, could be the explanation for unusually low viscosities whose values have not been found to be compatible with those usually provided by polymers that require a rod-shaped conformation, such as electrolytes in aqueous solutions. The hydrophobic interactions between the methyl groups of rhamnose scraps could explain ulvan behavior in aqueous solution [185]. The polysaccharide extraction and purification procedure could influence the viscoelastic properties of ulvan solutions, depending on the molecular weight distribution of the extracts obtained by different methods [169,185,186].

Ulvan, sulphated polysaccharide of green algal origin, has been shown to have a wide range of biological properties and could contribute to some beneficial health effects via algae consumption [171]. 

Some studies reported that the presence of sulphate groups could be interpreted as a determining factor in ensuring the antithrombotic activity of ulvan by promoting the binding of both specific and unspecific SPs to the proteins involved in the intrinsic coagulation pathway [171], but with not yet fully elucidated mechanisms [187]. 

Due to its chemical resemblance to mammalian heparinoid compounds, the ulvan’s anticoagulant activity was the most anticipated biological property. El-Baky et al., 2009, shows that SPs extracted from *Ulvales* spp. have been reported to have significant antithrombotic and anticoagulant activities [156].

#### 2.3.3. Ulvan-Based Hydrogels as Delivery Systems 

Taking into consideration the above biological properties of ulvan, some research has been focused on the feasibility of using ulvan as a polymeric matrix for biomedical applications with the development of hydrogels for tissue and cell engineering, also drug delivery systems. Among the various investigated areas, the biomedical field is the most natural application for hydrogels due to the important role of water in maintaining metabolic and electrolytic activities in living organisms. Furthermore, the hydrogels’ affinity and porosity could make these materials suitable for perfect integration into the tissues of living organisms, being a therapeutic source of bioactive compounds.

Also, numerous studies have reported the use of hydrogels in tissue engineering and drug/cell delivery system applications [188,189], and so far these hydrogels, particularly in the nanogel form, are the systems of choice for controlled and targeted drug delivery for local or systemic therapies [190]. 

Thereby, in tissue engineering, hydrogels have been investigated as physical supports for cell adhesion and/or encapsulation to restore tissues and organ functions [191,192], due to the biocompatibility, biodegradability, adhesive, and hydrophilic actions. In addition, the hydrogel applications emerge from the affinity of the water and porosity of these materials, which allows the cellular accommodation and the diffusion of the dissolved substances within the polymer network. 

In addition, some authors have shown that hydrogels could minimize irritation of host tissues due to their soft consistency and mechanical properties similar to those of natural extracellular matrices [193]. In order to optimize tissue regeneration, Metters and Lin, 2007, reported that the rate of hydrogel degradation in tissue engineering applications should match the cell growth and proliferation [194].

Regarding the drug delivery systems, Metters and Lin, 2007, showed that the rate of hydrogel degradation is used to modulate the pharmacokinetics and availability of encapsulated drugs, in agreement with the chemical structure of the polymeric materials. The crosslinking bonds supporting hydrogels are decisively affecting the biocompatibility and biodegradability of these systems [194]. 

Morelli and Chiellini, 2010 established a method for the preparation of chemically crosslinked ulvan-based hydrogels by UV light photopolymerization [176]. Morelli and Chiellini, 2010 and Pitarresi et al., 2003, reported that this method by light irradiation, was selected as a rapid, easy, and safe method since it did not require toxic catalysts and crosslinkers that are being used in the traditional methods [176,195]. 

**Figure 7:**
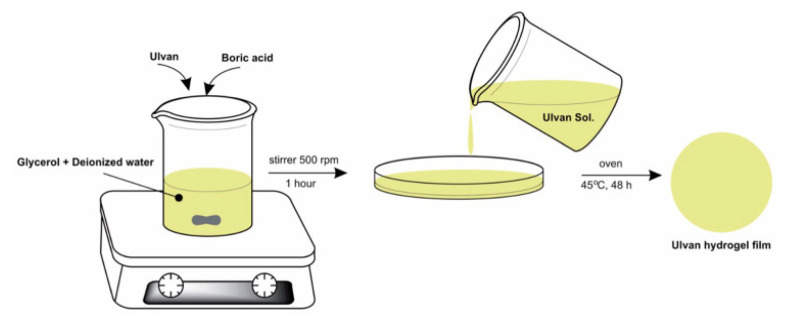
Method of ulvan hydrogel films obtaining (adapted from Sulastri et al., 2021, [196]).

Another approach for preparing the ulvan hydrogel films was developed by Sulastri et al., 2021 [196], by a simple method, through ionic crosslinking with boric acid and adding glycerol as a plasticizer, Figure 7. The obtained films showed that the different concentrations of ulvan in the formula affects the characteristics of the hydrogel film, can reduce hydroxyl radicals, and inhibit Gram-positive and Gram-negative bacteria (*Staphylococcus aureus*, *Pseudomonas aeruginosa*, *Escherichia coli*, and *Streptococcus epidermidis*). The obtained films could have potential for wound dressing material applications based on the biopolymer characteristics with antioxidant and antimicrobial activities [196].

Ulvan-based hydrogels, respectively UMA-based (ulvan methacrylate) hydrogels, conditioned as 3D polymeric networks were developed for a different application, bone tissue regeneration. Dash et al., 2014, reported that the enzymatic deposition of some apatite crystals led to a homogeneous distribution on the scaffold surfaces and represents one of the processes that could elicit the osteoblast cells proliferation and differentiation [197].

One promising method for preparing a bone regeneration substrate was described by Toskas et al., 2012 based on an ionic crosslinked ulvan scaffold [198]. The group developed a hybrid hydrogel as an ionically stabilized polyelectrolyte complex by the interactions between the negative charges of ulvan and positive charges of chitosan. By optimizing the ulvan/chitosan ratio, a nanofibrous structure was obtained, the biocompatibility being investigated in an osteoblast cell culture. The results of this research suggest that these ulvan/chitosan hydrogels may promote the attachment and proliferation of osteoblasts and could be promising scaffolds for bone tissue regeneration and recovery [198]. 

There has been a growing interest in the injectable hydrogel development due to the superior benefits of these systems compared to preformed hydrogels [193]. By exposing a precursor polymeric solution to some physiological stimuli, such as ionic strength, temperature, and pH, 3D cross-linked hydrogel polymer networks, were obtained, in situ. 

The enzymatic system for hydrogel preparation, represents a promising method to obtain safe materials. Morelli et al., 2016a, 2016b, reported a method in which ulvan has been modified with hydroxyphenyl units as specific substrates for horseradish peroxidase (HRP) in a conventional enzymatic system. Prior to the gelation process, these hydrogel systems could be used in tissue engineering and drug delivery systems as the cells or drugs could be homogeneously incorporated into the polymer solution [199,200]. 

An advantage of the injectable hydrogels, emphasized by Overstreet et al., 2012, could be the administration by simple and minimally invasive procedures, thus reducing the risks of surgical complications usually associated with the implantation of preformed hydrogels [201]. The liquid characteristics of the injecting solution allows the complete penetration of any defect areas whose irregular shapes could be perfectly adapted through the 3D polymeric network formed in situ [202].

Thermogelling systems of the ulvan-based gels, suitable for biomedical purposes are poly[N-isopropylacrylamide) (p(NIPAAm)], characterized by LCST (lower critical solution temperature) values below 37 °C taking into account the need to gelify in contact with human physiological conditions. These gelling systems represent a class of materials that exhibit a phase transition from liquid to solid above a critical temperature defined as the LCST and acceptable biocompatibility [203]. Klouda and Mikos, 2008, reported that the gelling process takes place through a mechanism of structural rearrangement of the polymer chains, in solution, leading to the formation of cross-linked physical bonds, stabilized by hydrophobic interactions.

#### 2.3.4. Ulvan-Based Polymeric Materials

In the development of ulvan-based biomedical devices, only few research papers addressed particularly the development of two-dimensional structures, such as coatings for antimicrobial applications and membranes for wound dressings (Table 4).

Other biomedical devices based on ulvan are the ulvan-based nanofibrous membranes obtained by the electrospinning technique and reported by [205]. In this paper, hydrophilic polymers, poly(vinyl alcohol) (PVA), and poly(ethylene oxide) (PEO) were mixed with polysaccharides to help spin the ulvan solution and to improve the mechanical integrity of the achieved membrane. To stabilize the nanofiber structure, a gelling system composed by boric acid/calcium ions was added to the polymer mixture. The integrity of the resulting membrane was enhanced by the ionic interactions, mediated by calcium ions, that occur between the borate esters formed on ulvan and PVA chains [205]. These preliminary mechanical and biological characterizations suggested its suitability for drug delivery systems applications and wound dressing.

In the field of biomedical coatings, the antiadhesive properties of ulvan were exploited in order to inhibit biofilm formation. The hydrophilic polysaccharides form a hydration layer on the surface, which acts as a physical barrier and inhibits cell adhesion to the surface [210]. In the context of increased antibiotic-resistant microbial strains, ulvan-based coatings are able to prevent bacterial contamination of medical devices and, therefore represent a promising alternative. This non-stick coating activity is also an effective strategy for preventing microbial biofilm deposition on medical devices [206]. The anti-adhesive properties of various ulvan coatings immobilized on silicone substrates have also been explored by the same authors [207]. 

Ulvan antibacterial properties have led scientists to study the immobilization on conventional poly (vinyl chloride) (PVC) based medical devices, [208] and the preliminary results showed a successful ulvan covalent conjugation on PVC surfaces via the urethane bonds formation.

A polymeric vehicle for the release of dexamethasone, used as a model of a hybrid particle-based drug, physically stabilized by ionic interactions of ulvan with chitosan, have been tested by Alves et al., 2012a. This particle formulation-based polymeric vehicle was incorporated into a 3D poly-dl-lactic acid (PDLLA) tissue scaffold to combine the PDLLA mechanical properties with ulvan, as a polymeric vehicle for drug delivery systems. The ulvan particles were incorporated into the PDLLA matrix, which led to a scaffold adapted for bone regeneration with mechanical and morphological properties [209]. These ulvan-enriched scaffolds showed the positive effect of the polysaccharide on cell viability and higher cytocompatibility when compared to simple PDLLA supports. The controlled release of dexamethasone, from the hybrid scaffold, indicates the suitability of the system developed for use in various biomedical applications [210].

The similarity with mammalian glycosaminoglycans could be exploited as pharmaceutical product delivery for the treatment of various conditions, for example the treatment of musculoskeletal disorders [211,212]. 

Treatment of skin pathologies, such as aging and the associated effects, could also benefit from ulvan bioactive properties which are directly correlated to the rhamnose moieties ubiquitous in the ulvan backbone structure [213,214]. 

Furthermore, due to the ulvan recognition by the hepatocyte membrane receptors Massarelli et al. 2007, advanced the idea of potential applications in areas such diagnostic or therapeutical approaches [215].

Moreover, Lahaye and Robic 2007, observed that ulvan can be used in metal poisoning therapy or for targeted radioactive treatment of tumors, as already proposed for another bioactive compound, carrageenan, extracted from red macroalgae [121,216].

To conclude, the recent studies reviewed constitute the basis for the selection of ulvan for various medical applications over other polymers. Nevertheless, there are still some challenges, such as the efficiency of the target delivery, the control over the release rate, fitting into the required therapeutic window, and the flexibility of routes of administration (e.g., oral instead of intravenous). While the task of translating basic ulvan research into practical achievements seems insurmountable, this polysaccharide applicability as drug delivery systems has great potential and versatility. 

## 3. Conclusions

The sulfated polysaccharides, namely carrageenan, fucoidan, and ulvan, with the various biological and structural properties, supported by the specific chemical structure, have contributed to the recent advancements in drug delivery strategies.

Taking into consideration the advantages of scientific knowledge, the gathered research can now be directed to practical applications in an attempt to reduce the gap between scientific research and industrial application.

However, the uncertainty regarding structures and the difficulties in extraction are definitely the strongest limitations regarding the applications proposal, thus slowing the progression of these materials to more advanced therapeutic solutions. Additionally, advanced toxicity assays will be needed to evaluate the possibility of using the materials in drug delivery approaches.

This review suggests that marine bioactive compound-derived polysaccharides are good candidates for future biomedical applications, in particular the development of innovative systems for drug delivery and tissue engineering approaches.

In conclusion, the interest in using marine bioactive compounds, in particular, algal polysaccharides, as biomedical vehicles for drug delivery systems has increased steadily and applications of these polysaccharides at the level of cell targeting will certainly be further explored in the future, as therapeutic approaches to local and systemic diseases might be envisaged.

## Figures and Tables

**Table 1:** Common seaweed species in the Black Sea [4].

Green Algae	Red Algae	Brown Algae
*Bryopsis hypnoides*	*Apoglossum ruscifolium* *	*Cladostephus spongiosus* *
*Bryopsis plumosa*	*Callithamnion corymbosum*	*Corynophlaea umbellata* *
*Chaetomorpha aerea*	*Callithamnion granulatum*	*Cystoseira barbata*
*Chaetomorpha linum*	*Ceramium arborescens*	*Cystoseira crinita* *
*Cladophora albida*	*Ceramium ciliatum*	*Dictyota dichotoma* *
*Cladophora dalmatica*	*Ceramium deslongchampsii*	*Dictyota fasciola* *
*Cladophora laetevirens*	*Ceramium diaphanum*	*Dictyota spiralis* *
*Cladophora liniformis*	*Ceramium virgatum*	*Ectocarpus siliculosus*
*Cladophora sericea*	*Chondria capillaris* *	*Nereia filiformis* *
*Cladophora vagabunda*	*Chondria dasyphylla* *	*Padina pavonica*
*Cladophoropsis membranacea* *	*Coccotylus truncatus* *	*Scytosiphon lomentaria*
*Codium vermilara* *	*Corallina elongata* *	*Spermatochnus paradoxus* *
*Ulva clathrata*	*Corallina officinalis*	*Sphacelaria cirrosa* *
*Ulva flexuosa*	*Dasya baillouviana* *	*Stilophora tenella* *
*Ulva (Enteromorpha) intestinalis*	*Dasya hutchinsiae*	*Zanardinia typus* *
*Ulva linza*	*Gelidium crinale* *	
*Ulva prolifera*	*Gelidium spinosum* *	
*Ulva rigida* syn. *Ulva lactuca*	*Gracilaria dura* *	
	*Gracilaria gracilis*	
	*Grateloupia dichotoma* *	
	*Haliptilon virgatum* *	
	*Jania rubens*	
	*Laurencia coronopus* *	
	*Laurencia obtusa* *	
	*Lomentaria clavellosa*	
	*Nemalion helminthoides* *	
	*Nitophyllum punctatum* *	
	*Osmundea pinnatifida*	
	*Palisada perforata*	
	*Peyssonnelia dubyi*	
	*Phyllophora crispa*	
	*Phymatolithon lenormandii*	
	*Polysiphonia elongata*	
	*Polysiphonia fucoides*	
	*Polysiphonia subulifera*	
	*Porphyra leucosticta*	

* absent or rare on the Romanian coast.

**Table 4:** Ulvan Based Biomedical Devices.

Biomedical Device	References
Membranes	[204]
	[205]
Coatings	[206]
	[207]
	[208]
Micro/nanoformulations	[209]

## Data Availability

Not applicable.

## Abbreviations

ROS	Reactive oxygen species
FTIR	Fourier-transform infrared spectroscopy
TNF-alpha-induced	tumor necrosis factor
HeLa	human cervical cancer cells
NDF	neutral detergent fiber
LDL	low-density lipoprotein
CVDs	cardiovascular diseases
SPs	sulphated polysaccharides
GAGs	glycosaminoglycans
UMA	ulvan methacrylate
LCST	lower critical solution temperature
PVA	poly(vinyl alcohol)
PEO	poly(ethylene oxide)
PVC	poly (vinyl chloride)
PDLLA	poly-dl-lactic acid
